# Differential response of patient-derived primary glioblastoma cells to environmental stiffness

**DOI:** 10.1038/srep23353

**Published:** 2016-03-21

**Authors:** Thomas James Grundy, Ellen De Leon, Kaitlyn Rose Griffin, Brett William Stringer, Bryan William Day, Ben Fabry, Justin Cooper-White, Geraldine Margaret O’Neill

**Affiliations:** 1 Children’s Cancer Research Unit, Kids Research Institute, The Children’s Hospital at Westmead, Westmead, 2145, New South Wales, Australia; 2 Discipline of Paediatrics and Child Health, The University of Sydney, Sydney, 2000, New South Wales, Australia; 3 Brain Cancer Research Unit & Leukaemia Foundation Research Unit, QIMR Berghofer Medical Research Institute, Brisbane, 4006, Queensland, Australia; 4 Department of Physics, University of Erlangen-Nuremberg, Germany; 5 Tissue Engineering and Microfluidics Laboratory, Australian Institute for Bioengineering and Nanotechnology, University of Queensland, St. Lucia, Queensland, Australia

## Abstract

The ability of cancer cells to sense external mechanical forces has emerged as a significant factor in the promotion of cancer invasion. Currently there are conflicting reports in the literature with regard to whether glioblastoma (GBM) brain cancer cell migration and invasion is rigidity-sensitive. In order to address this question we have compared the rigidity-response of primary patient-derived GBM lines. Cells were plated on polyacrylamide gels of defined rigidity that reflect the diversity of the brain tissue mechanical environment, and cell morphology and migration were analysed by time-lapse microscopy. Invasiveness was assessed in multicellular spheroids embedded in 3D matrigel cultures. Our data reveal a range of rigidity-dependent responses between the patient-derived cell lines, from reduced migration on the most compliant tissue stiffness to those that are insensitive to substrate rigidity and are equally migratory irrespective of the underlying substrate stiffness. Notably, the rigidity-insensitive GBM cells show the greatest invasive capacity in soft 3D matrigel cultures. Collectively our data confirm both rigidity-dependent and independent behaviour in primary GBM patient-derived cells.

Early and widespread invasion of glioblastoma (GBM) makes total surgical resection unlikely and the probability of recurrence high^1^. Despite the implementation of new therapies, invasion remains a major impediment to curing GBM. Based chiefly on advances in the breast cancer research field, it is increasingly realized that mechanical cues within the external tissue environment play a major role in invasive capability^2^,^3^,^4^,^5^,^6^,^7^,^8^. Initial studies suggested that GBM cell invasion is inhibited on soft brain-like matrices and increases with higher matrix rigidities, thus suggesting that GBM are rigidity-sensitive^9^,^10^,^11^. However, these findings are contrasted by newer analyses of primary patient-derived GBM lines that were shown to be rigidity-independent^12^,^13^. It is now realized that GBMs represent molecularly and genetically distinct subclasses^14^,^15^,^16^,^17^ and the majority of commonly used, repeatedly cultured GBM lines (such as those used in the initial studies of rigidity response) are of the Mesenchymal subclass^14^. Thus, in order to establish whether all primary patient-derived lines are indeed rigidity-independent or whether there is variation between patients, it is important to analyse a range of primary lines. In the present study we compare the rigidity dependent migration behaviour of 5 primary patient-derived cell lines.

In common with other solid tumours, GBM tumours are also subject to different mechanical forces. The matrix proteins secreted by GBM counteracts expansion of the tumour tissue and thereby increases mechanical forces on the tumour^18^,^19^. For rapidly growing GBMs and at later stages in disease progression, forces are increased on the whole brain as the rigid skull prevents tissue expansion^20^. The natural ECM of the brain tissue and in particular the ECM secreted by the GBM cells stiffens in response to the increasing pressure and the resulting tissue strain^21^. Importantly, glial cells do not migrate efficiently in the very soft brain tissue, and strain stiffening of the ECM improves glial cell migration: disabling this effect reduces GBM invasion^11^. Finally, GBMs stiffen in response to compressive pressure^21^. Therefore the natural course of the disease can induce mechanical forces and tumour stiffening. Moreover, brain tissue that is encountered by invasive GBM cells is mechanically heterogeneous with micro-regional stiffness values ranging from as low as 0.1 kPa to as high as ~10 kPa^22^. This mechanical heterogeneity is important in the regulation of normal brain biology^22^,^23^,^24^,^25^ and thus may also influence tumour cells as they invade different brain regions. In response to external mechanical forces, cells exert increasing Rho-GTPase dependent contractile force through their acto-myosin cytoskeleton^6^. This leads to greater traction forces on the surrounding extracellular matrix (ECM) and enhanced migration and invasion. The ability of cancer cells to proliferate and migrate on engineered substrates of defined rigidities is further reflected in their abilities to grow in specific tissue environments *in vivo*^26^.

The limitation that most commonly used cultured GBM lines are of the mesenchymal subclass^14^ is overcome by the isolation of primary patient-derived GBM lines and maintenance in serum-free, Glioma Neural Stem (GNS) media at low passage^27^,^28^. In the present study we reveal diverse rigidity-dependent responses in primary GBM lines.

## Results

### JK2, WK1, RN1 and PR1 cell morphology are regulated by substrate stiffness, but SJH1 cell morphology is stiffness insensitive

**Figure 1:**
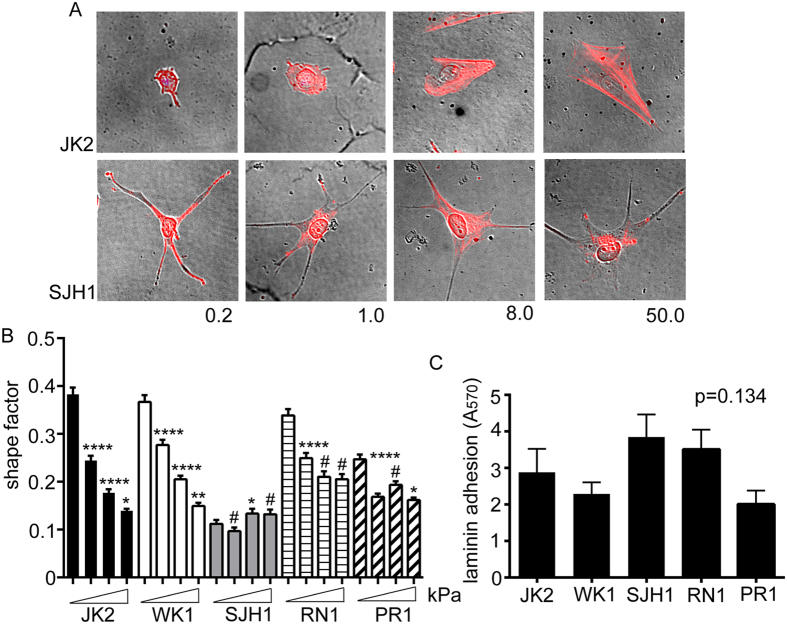
Rigidity-dependent morphological responses. (**A**) Bright field images of cells on PAM gels of the indicated Elastic moduli, overlayed with images of the same cells immunostained with fluorescently tagged phalloidin to detect F-actin. (**B**) Mean cell shape factor (4πA/P^2^; A = area, P = perimeter) for the indicated cells grown on PAM gels of increasing stiffness (0.2, 1.0, 8.0, 50 kPa). N > 198 cells analysed per condition, per cell line. *p < 0.05, **p < 0.01, ****p < 0.0001, ^#^not significant, one-way ANOVA with Tukey’s post-comparison. Statistical comparisons shown indicate differences between increasing stiffness (0.2 versus 1.0, 1.0 versus 8.0 and 8.0 versus 50). (**C**) Adhesion of the indicated cell lines to laminin-coated wells. Values shown are the A_570_ and represent the average of 3 individual repeats. Error bars on all graphs show the SEM. There was no significant difference in adhesion to laminin between the three cell lines, (p = 0.134) one-way ANOVA.

We compared the responses of 5 primary GBM lines (JK2, SJH1, WK1, RN1 and PR1). Cells were grown on polyacrylamide hydrogels (PAM gels) of defined rigidity, corresponding to the reported range of Young’s modulus (*E*) values for brain tissue (0.2, 1.0 and 8.0 kPa)^22^ and 50 kPa (upper physiological limit of tissue stiffness^29^). Gels were coated with matrigel to provide an adhesive ligand for the cells. Importantly, gel stiffness does not affect matrix coating density^30^. The JK2, WK1, RN1 and PR1 cells were more rounded on the softest gels. Each of these 4 lines exhibited increased spreading on stiffer gels but the RN1 and PR1 lines showed limited increases on the hardest gels. Both JK2 and WK1 showed evidence of arced membranes lined with polymerised actin on the stiffest (50 kPa) substrates (Fig. 1A). By contrast, SJH1 cell morphology was highly similar irrespective of the underlying rigidity (Fig. 1A). Assessment of mitotic events throughout the time-lapse period revealed that there were no significant differences between the cell lines (Supplementary Figure 2). Quantification of cell shapes confirmed that JK2 and WK1 cells progressively lost their rounded phenotypes as the matrix rigidity increased, RN1 and PR1 spreading peaked at 8.0 kPa and 1.0 kPa, respectively, and the shape of SJH1 cells was unaffected (Fig. 1B). Importantly, the lack of response by the SJH1 cells was not due to differences in adhesion to the matrigel-coated PAM gels, as there was no difference in adhesion between the cell lines to laminin, the chief component of matrigel (Fig. 1C).

### Cell speed is differentially regulated by substrate stiffness

**Figure 2:**
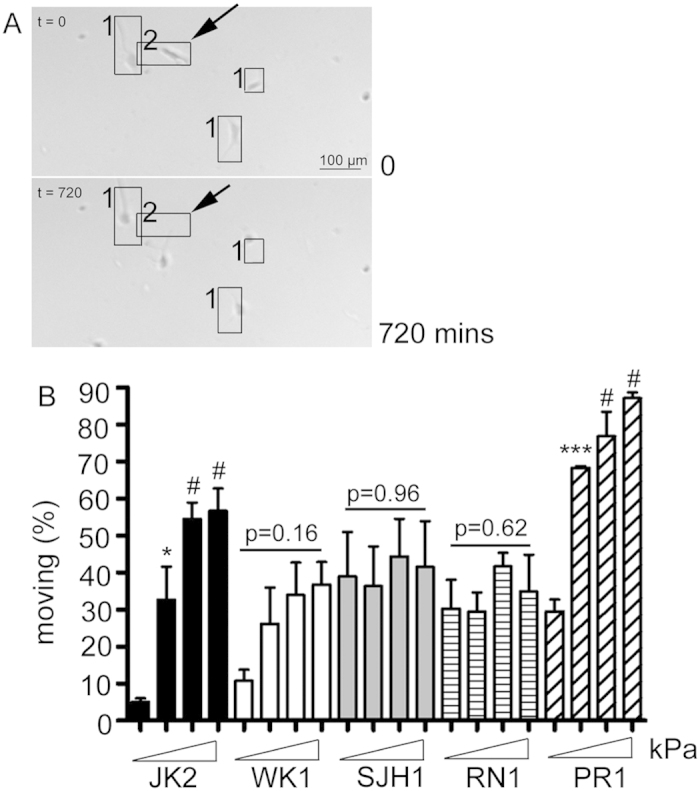
Percentage of motile cells. (**A**) Cells were categorised as either not moving (for example cells labelled ‘1’ in the image) or moving (cells labelled ‘2’) depending on whether the cell nucleus remained contained within the boxed region that encompassed the entire cell at time 0 for the entire 720 minute viewing period, or moved out of the box. (**B**) The percentage of moving cells from each cell line on the indicated PAM hydrogels. Columns show the average of data from 3 independent repeats. Error bars show the SEM. *p < 0.05, ***p < 0.001, ^#^not significant one-way ANOVA with Tukey’s post-comparison. Statistical comparisons shown indicate differences between increasing stiffness (0.2 versus 1.0, 1.0 versus 8.0 and 8.0 versus 50). There was no significant difference in the percentage of motile cells in the WK1, SJH1 and RN1 cells (p values indicated on graph), one-way ANOVA.

We next investigated the effect of substrate rigidity on cell migration. When analysing time-lapse movies of cells grown on PAM gels with different stiffness, it was noted that not all cells in the populations were migratory. Thus, cells were scored as moving or non-moving (as described in the materials and methods) (Fig. 2A). This revealed that very few JK2 cells were motile on the softest gels (5.0% ± 0.96), suggesting that this soft environment is inhibitory to JK2 movement (Fig. 2B). With increasing substrate stiffness, more JK2 cells were migratory with little difference observed between the 8.0 and 50 kPa gels (55% ± 4.4 versus 57% ± 6.1, respectively). Similar to the JK2 cells, the percentage of motile PR1 cells was significantly increased on the 1.0kPa gels, with no further significant increases on the subsequent gel rigidities (Fig. 2B). The percentage of motile cells was unaffected by substrate stiffness in the WK1, SJH1 and RN1 cells (Fig. 2B).

**Figure 3:**
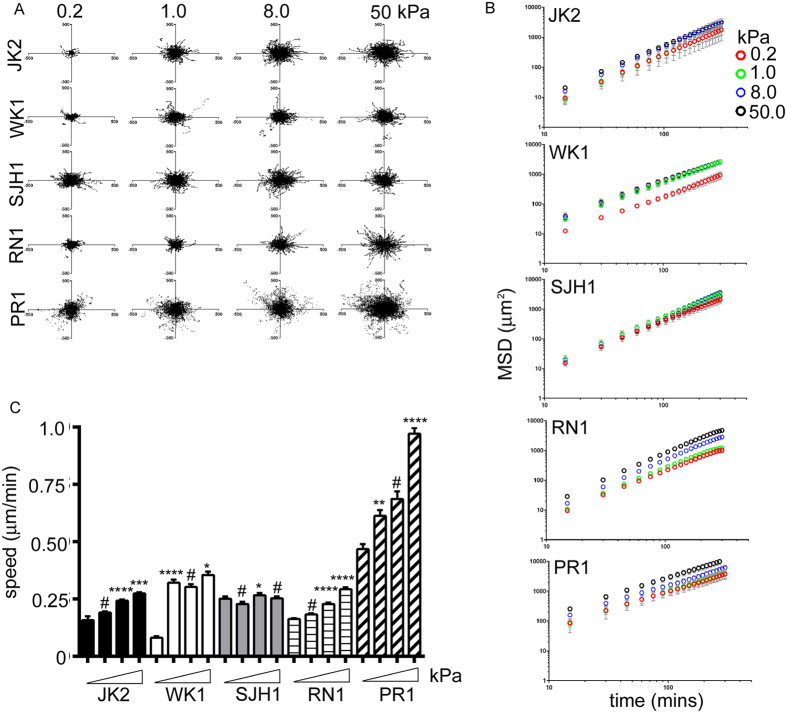
Cell speed in response to external substrate stiffness. **(A**) Migration traces for cell lines under the indicated conditions. (**B**) MSD calculated from cell trajectories on 0.2 (red), 1.0 (green), 8.0 (blue) and 50 kPa (black) PAM gels. (**C**) Average speed of the indicated cell lines on the different stiffness PAM gels. Note that speed was only calculated from the population of cells categorised as moving. Data are pooled from 3 independent repeats, n > 150 cells tracked per condition, with the exception of the JK2 cells on the 0.2kPa gels, in which only low numbers of cells were motile (n = 34). Error bars show the SEM. *p < 0.05, ***p < 0.001, ****p < 0.0001, ^#^not significant, one-way ANOVA with Tukey’s post-comparison. Statistical comparisons shown indicate differences between increasing stiffness (0.2 versus 1.0, 1.0 versus 8.0 and 8.0 versus 50)

We next analysed the speed of the motile cells in response to substrate rigidity. Comparison of wind-rose plots of tracked cells indicated that migration increased in response to increasing substrate stiffness in JK2, WK1, RN1 and PR1 cells (Fig. 3A). However, the migration paths traced by the motile SJH1 cells and their Mean Squared Displacements (MSD) were indistinguishable for different substrate rigidities (Fig. 3A,B). Interestingly, while the MSD of the JK2, RN1 and PR1 cells monotonically increased with substrate stiffness, the MSD of WK1 cells increased only for low stiffness values but then levelled off and coincided on 1.0, 8.0 and 50 kPa PAM gels (Fig. 3B). Individual cell speed data for all cell lines and matrix stiffnesses mirror and confirm the findings from the MSD and the wind-rose plots (Fig. 3C).

### Actin organization and substrate stiffness

**Figure 4:**
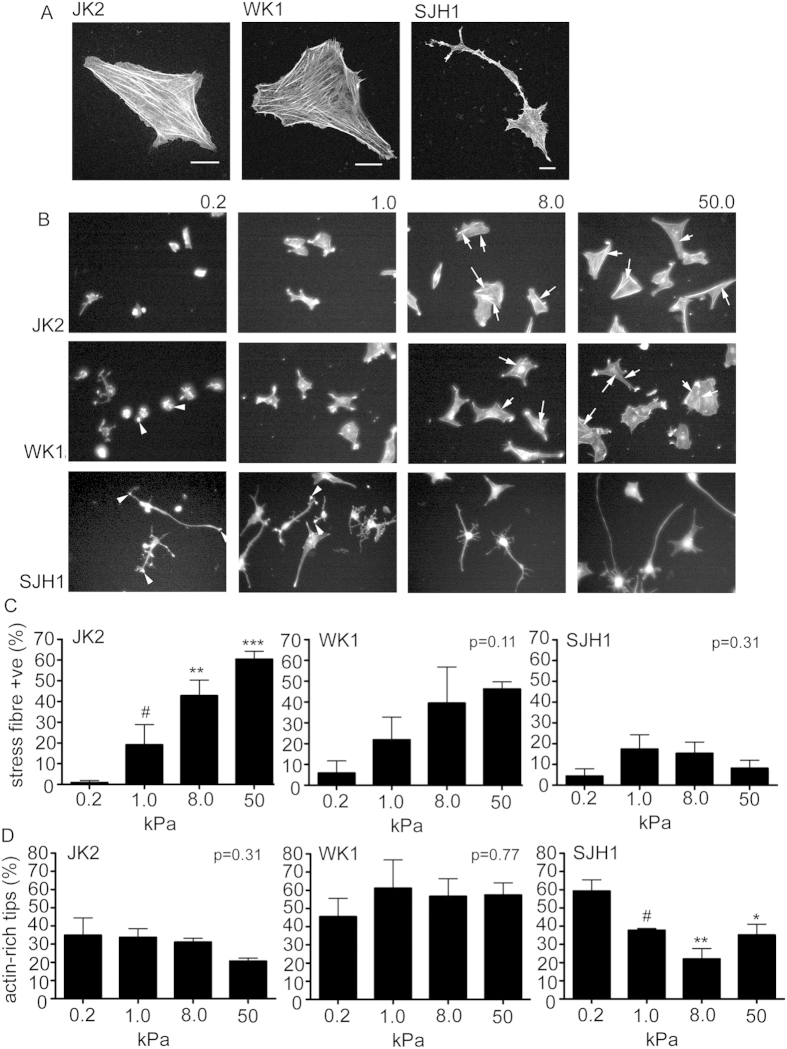
Actin filament organization. (**A**) Confocal images of cells grown on glass coverslips showing the actin filament organization. Scale bars 10 μm. (**B**) Epifluorescent images of cells grown on poly-acrylamide hydrogels of the indicated rigidities. Arrows point to examples of stress fibres (see JK2 and WK1 cells on 8.0 and 50.0 kPa gels). Arrow heads point to examples of actin-rich tips (see WK1 0.2kPa and N 0.2 and 1.0 kPa). (**C**) Percentage of cells that display actin stress fibres. (**D**) Percentage of cells that display actin-rich tips. Data represent the average percentage from 3 independent replicates per cell line, per substrate rigidity. Error bars show the SEM. *p < 0.05, **p < 0.01, ***p < 0.001, ^#^not significant, one-way ANOVA with Tukey’s post-comparison. Statistical comparisons shown indicate comparison with phenotype on 0.2kPa gels. Statistical comparisons shown indicate differences between increasing stiffness (0.2 versus 1.0, 1.0 versus 8.0 and 8.0 versus 50). All other data shown were not significantly different (p values indicated on graphs), one-way ANOVA.

Mechano-responsive cells form filaments of actin polymers (stress fibres) in response to increasing external mechanical force. Given that the SJH1 cells were insensitive to changes in the external substrate rigidity, we questioned whether these cells form stress fibres. Strikingly, even when plated on matrigel-coated glass (many orders of magnitude stiffer than the 50 kPa PAM gels), few SJH1 cells displayed stress fibres (Fig. 4A). Comparison of the rigidity insensitive SJH1 with two examples of rigidity sensitive lines (JK2 and WK1) revealed that the rigidity-sensitive lines displayed prominent stress fibres on a glass surface (Fig. 4A). Interestingly, comparison of stress fibre formation between the JK2 and WK1 cells suggested that the JK2 cells exhibited increasing stress fibre formation in response to external substrate stiffness, but this effect was less pronounced in the WK1 cells (Fig. 4B). Quantification of the percentage of cells displaying stress fibres confirmed that stress fibre formation in JK2 cells is significantly increased with external substrate stiffness (Fig. 4C). By contrast, although there is a trend for increased stress fibres in the WK1 cells – and there are significantly more stress fibre positive cells on the 50 kPa versus the 0.2 kPa substrates (*p* < 0.01 Students’ *t*-test) - large variations in stress fibre formation within cell populations grown on 1.0 and 8.0 kPa gels means that the increase in stress fibre formation did not reach a significance level of p < 0.05 (Fig. 4C). Agreeing with the limited stress fibre formation seen in SJH1 cells on glass substrates (Fig. 4A), there was limited stress fibre formation in SJH1 cells on any of the different stiffness gels, with no significant changes between gels (Fig. 4C). However, the SJH1 cells frequently exhibited actin-rich tips at the ends of long, thin membrane processes (Fig. 4D). These tips were significantly reduced on the 8.0 and 50 kPa gels, with a prominent dip in the percentage of cells with actin-rich tips on the 8.0 kPa gels (Fig. 4D).

### Rigidity-insensitive N cells invade further

**Figure 5:**
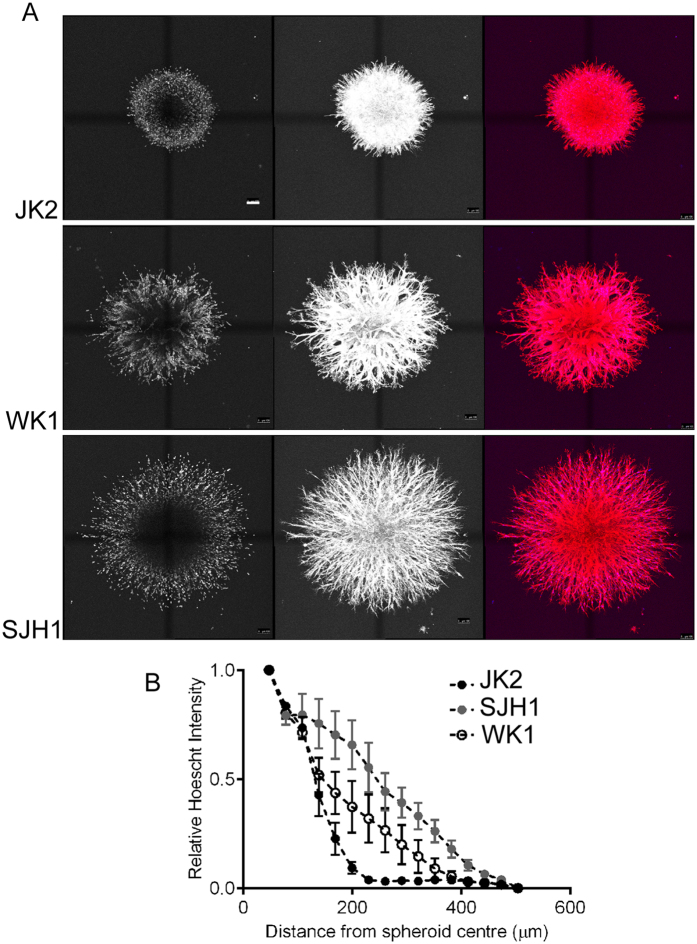
Comparison of 3D invasion. **(A**) Representative confocal images (maximum projection) of the indicated multicellular spheroids embedded in matrigel and allowed to invade for 48 hours. Spheroids were then fixed and immunostained with Hoescht and fluorescently-tagged phalloidin to detect nuclei and actin respectively. Final column shows the merged image of the nuclei and actin stains. Scale bar = 100 μm. (**B**) Quantification of invasion by the indicated multicellular spheroids. Data are the average of >7 individual spheroids, from 3 individual experiments. Error bars represent SEM. JK2 versus SJH1: *P* < 0.0001; WK1 versus SJH1: NS; JK2 versus WK1: *P* = 0.0056, two-way ANOVA.

Collectively, the data suggest that both WK1 and JK2 cell migration is inhibited in soft environments, while SJH1 cell migration is insensitive to local substrate stiffness, suggesting that SJH1 cells may invade further in soft 3-dimensional environments. To test this, we compared the invasive behaviour of the three cell subtypes embedded in soft 3D matrigel. In order to model the collective and individual cell invasion that cancer cells exhibit *in vivo*, we analysed multicellular spheroids embedded in matrigel. Each cell type exhibited collective strand invasion into the surrounding matrigel, and there was little evidence of any single cell invasion (Fig. 5A). The extent of invasion of each cell type was consistent with the rigidity-sensing migration behaviour. Thus, the JK2 cells that showed the greatest sensitivity to rigidity – both in terms of total motile population and speed of migration – invaded significantly less than either the WK1 or the N cells (Fig. 5B). Notably, SJH1 cell spheroids tended to invade more than the WK1 cells. This increase does not reflect changes in proliferation as the N cells grow more slowly than the other lines. Thus the rigidity-insensitive N cells invade further into soft 3D matrigel, than the WK1 and JK2 cells.

## Discussion

We have compared rigidity-dependent behaviour in 5 primary GBM cell lines. In line with earlier data showing that continually cultured lines exhibit rigidity-dependent migration^9^,^10^,^11^, we find that migration of 4/5 primary patient-derived lines was also restricted on the softest substrates that mimic healthy brain tissue. Conversely the SJH1 cells migrated efficiently on the soft brain-like environment, correlating with more extensive invasion into a soft 3D matrigel environment.

Similar to our data for the SJH1 cells, it was recently reported that another primary patient-derived cell line is rigidity independent^13^. The earlier reported cells represent the Classical subclass and it is interesting to consider whether the different rigidity-dependent responses might reflect sub-class specific behaviour. For example, molecular sub-typing of the SJH1 cells suggests that they are of the neural sub-class. The fact that they are insensitive to substrate rigidity is consistent with the ridigity-insensitive phenotypes of neurons which extend long neurites irregardless of the substrate rigidity^25^. Similarly, mesenchymal subclass cells (including the WK1 cells analysed in the present study) are most similar to astrocytes^14^, which fail to spread and generate actin stress fibres on soft substrates^25^. Given that neural cell lineages that correlate with distinct GBM subclasses display different mechano-sensitivity^14^,^24^,^25^, it is possible that differential rigidity responses may reflect these discrete neural cell lineages. Alternatively, it has previously been proposed that tumour location (cells from peritumoral versus tumour mass) correlates with rigidity-dependent responses^12^. Although the genetic subclass of the different tumour populations examined in this earlier study were not described, it would be of interest to determine whether these differences may reflect underlying genetic subclasses from different areas of the tumour. Importantly, differential rigidity responses could provide distinct migratory responses between patient tumours.

Analysis of time-lapse movies highlighted differences in the intrinsic migratory capacity of cells within each cell line. While the percentage of migratory JK2 and PR1 cells increased in response to increasing external substrate rigidity, the total percentages of SJH1, WK1 and RN1 cells were unchanged. As the migration analyses were performed in the absence of any externally imposed chemotactic or haptotactic gradient, the data reveal the intrinsic migratory capacity of the cells. We could detect no association between cell morphology and migratory capacity, thus, it is currently not known why some cells in the population are migratory while others remain essentially stationary. Potentially the differences in intrinsic migratory capacity may represent molecular heterogeneity within the cultures^31^. Alternatively, the average migration speed of a cell population (inter-individual) is suggested to be stable over time, but the speed of individual cells (intra-individual) is not consistent following cell division^32^. Thus, additional factors, such as epigenetic modifications, may be critical for determining the velocity of individual cells.

Matrigel has been demonstrated to be a mechanically soft gel in a number of studies, with the average value reported to be ~0.4 kPa^6^,^33^,^34^. In the present study we therefore used matrigel to assess the invasion of 3 GBM lines in which we had defined rigidity-responses, through a mechanically soft 3D environment. Since both collective and individual invasion are evidenced in multi-cellular spheroids cultured in 3D matrices^35^,^36^, we assessed the invasion of multicellular GBM spheroids in soft matrigel cultures. Under these conditions, the three GBM lines exhibited collective strand invasion. Notably, the SJH1 cells were more invasive than either of the other cell lines, agreeing with the rigidity-insensitive phenotype of these cells.

In addition to changes in mechanical forces contained within the tumour as the disease progresses^11^,^21^, once the GBM cells escape the primary tumour mass they encounter regions with different tissue features. The invasive GBM typically disseminate via two discrete anatomical routes, either along myelinated nerve fibres in the soft white brain matter or along stiffer basement membranes surrounding blood vessels^37^,^38^. Thus, the mechano-response of the cells may be an important determinant of their route of dissemination. In addition to regulating cell migration and proliferation^26^, matrix rigidity can regulate a range of cellular processes including cell signalling^3^,^39^,^40^,^41^ and alternative splicing^42^. Our study has clearly demonstrated that primary patient-derived GBM cells display a range of rigidity sensing phenotypes. In future it will be interesting to explore the potential relationship between rigidity phenotypes and GBM sub-class. Given the heterogeneity of GBM tumours, such a study would require analysis of a large number of lines in order to confirm cause-and-effect between sub-class and rigidity response. More definitive proof could be obtained by instead manipulating the transcriptional programs that regulate the sub-classes and then testing rigidity response. Understanding the response of GBM cells to the external mechanical cues in the brain and how this may differ between different tumours holds great promise for identifying new therapeutic targets for GBM.

## Methods

### Cell lines

The GBM patient-derived cell lines JK2, SJH1, WK1, RN1 and PR1^27^,^43^,^44^ were established by the Brain Cancer Research Unit, QIMR Berghofer Medical Research Institute, Brisbane, Australia as previously described^28^. Patient tissue was collected following written informed consent and with human ethics committee approval from the Royal Brisbane and Women’s Hospital, Brisbane and the QIMR Berghofer Medical Research Institute and in accordance with the approved guidelines. Subtyping was performed as described^14^. Details of the patients from whom the lines were derived and cell line characteristics are shown in Supplementary Table S1.

### Cell culture maintenance and media

All cell lines were cultured and maintained in filter-capped tissue culture flasks coated with Matrigel solution (1% Matrigel growth factor reduced Basement Membrane Matrix LDEV-Free (v/v), DMEM High Glucose Pyruvate). Cell culture media for all cell lines consisted of KnockOut™ DMEM/F-12 supplemented with Recombinant Human EGF (20 ng/mL), Recombinant Human FGFb (10 ng/mL), Glutamine (20 mM/mL), Heparin (20 ng/mL), Penicillin/ Streptomyocin (100 U/mL) and StemPro Neural Supplement (20 ng/mL). Cells were maintained at 37 °C and 5% CO_2_. Examples of the morphology of early passages of each cell line is shown in Supplementary Figure 1. For all experiments it was confirmed that cells maintained these morphologies and culture passages were limited to a maximum of 35. All experiments were performed on lines representing different passage numbers to ensure that data did not reflect passage number. Each line was routinely sub-cultured as follows: JK2 cells were sub-cultured at a dilution of 1/5 every 5 days; WK1 cells were sub-cultured 1/5 every 6–7 days; RN1 and PR1 cells 1/3 every 4 days; and SJH1 cells were sub-cultured 1/3 to 1/4 every 10 to 14 days.

### Polyacrylamide (PAM) hydrogels

Easy-coat polyacrylamide (PAM) hydrogels of defined Young’s modulus (*E*) in 35 mm Petrisoft (plastic-bottom) and Softview (glass-bottom) cell culture dish formats were purchased (Matrigen, CA USA). Petrisoft dishes were used for bright-field based assessment of migration and morphology experiments, and softview for confocal microscopy. PAM hydrogels were coated with a thin layer of Matrigel solution at room temperature (RT) for 1 hr under sterile conditions and then used immediately. Cells were seeded onto gels at densities that yielded single cells that were not touching other cells.

### Generation of multi-cellular spheroids

In order to generate spheroids, cells were seeded on 0.8% agarose coated 96-well plates in media and incubated at 37 °C for 5 days. To facilitate comparison between cell lines with different proliferation rates, initial experiments were carried out to determine the cell numbers required to provide equivalently sized spheroids at the time of initial plating. Thus the following numbers of cells were routinely plated to generate spheroids: 15,000× JK2, 20,000× WK1 and 20,000× SJH1 cells. Spheroids were washed in PBS prior to embedding in 1.7mg/ml collagen gels. After 1 hour of gel polymerisation at 37 °C, complete media was added and spheroids were then incubated for 48 hours.

### Immunofluorescence

Cells cultured on matrigel-coated PAM hydrogels were fixed in 4% PFA (v/v), then incubated with Quenching Buffer for 10 minutes (0.15M Glycine in PBS), permeabilised for 5 minutes (0.2% Triton X-100 (v/v), 0.5% Bovine Serum Albumin (w/v) in PBS) and incubated in Blocking Solution for 1 hr. Subsequently, cells were stained with TRITC Phalloidin and DAPI using standard protocols. Spheroids embedded in 3D collagen gels were fixed with 4% PFA, and treated with 0.15 M Glycine in PBS to quench background fluorescence. Gels were then permeabilised in 0.2% Triton X-100 in PBS and blocked in PBS containing 1% BSA. Spheroids were then immunostained with TRITC-phalloidin and Hoechst Blue nuclear stain and stored in PBS until imaging.

### Cell Imaging

Epifluorescence and bright-field images were captured using an ORCA-AG ERG cooled CCD camera (Hamamatsu/ SDR Clinical Technology, Australia) coupled to an Olympus IX81 (Olympus, Tokyo Japan) inverted microscope maintained at 37 °C throughout live imaging experiments using an environmental chamber (Solent Scientific Limited, Fareham UK). Cells were viewed with the following objectives: Olympus 20× (0.45 N.A.) UPlanFLN dry objective and 4× (0.13 N.A.) LUCPlanFLN Objective. Filters for fluorescent imaging were: BP360-370/LP420 (DAPI); BP530-550/ BP575-625 (TRITC). Confocal microscopy imaging was performed with a Leica TCS SP5 Confocal System (Leica Microsystems, Wetzlar Germany) with either a 63× oil objective (used with glass slides) or a 63× water dipping objective. Collagen embedded and immunostained spheroids were imaged with the Leica TCS SP5 and a 10× air objective. Maximum projections and analyses were performed using Leica LAS software.

### Adhesion assay

Cell adhesion to fibronectin, collagen I, collagen IV, Laminin I, and Fibrinogen was analysed using CytoSelect 48-Well Cell Adhesion Assay (ECM Array, Colorimetric Format) assay kits (Cell Biolabs Inc., CA USA). The advised assay protocol was followed with exception that a larger number of cells than the recommended number was required and 9 × 10^5^ cells were used per cell line per well.

### Analysis of cell migration

For live imaging, media was replaced with CO_2_-Independent Media supplemented with Recombinant Human EGF (20 ng/mL), Recombinant Human FGFb (10 ng/mL), Glutamine (20mM/ml), Heparin (20 ng/mL), StemPro Neural Supplement (20 ng/mL) and Penicillin/ Streptomyocin (100 U/mL). Dishes were covered in Parafilm (Bemis, WI USA) and equilibrated at 37 °C for 20 minutes. Time-lapse microscopy was carried out on Parafilm-covered dishes over a 12 hour time period, with four fields of view (FOV) captured per dish at 15 minute intervals. Following image acquisition, cells were binned into moving and non-moving. Cells were classified as “moving” if the nucleus moved (for any period of time) outside a boxed region drawn to encompass the entire cell at t = 0. Cells in which the nucleus either never left the drawn box, or did not fully leave the box were classified as non-moving cells. Moving cells were then tracked using Metamorph 7.1 software. Data collected included the Cartesian coordinates of individual cells at each time point throughout an experiment, as well as the total distance travelled by each cell. Cell speed was calculated using the distance travelled of a cell between frames divided by the time period between frames (15 minutes). The Cartesian coordinates for each time period of each cell were used to calculate Mean Squared Displacement, as well as construct scatter plots.

### Image processing, measurement and statistical analysis

Image processing and cell measurements were carried out with Metamorph version 7.1 Image Analysis software (Molecular Devices, CA USA). Images for cell morphology were thresholded based on cell-background contrast, followed by automated definition of cell edge. Where this automated process was not possible, cell edges were traced by hand. Images were processed, with data pertaining to cell area, length, breadth, perimeter and shape factor collected. Analysis of spheroid invasion was performed by concentric circle analysis with ImageJ software. A series of concentric circles (16 circles, inner radius of 47.3 μm, outer radius of 473 μm) was overlayed on spheroid images, and the average Hoechst pixel intensity along the perimeter of each circle was calculated. For each spheroid, pixel intensities were normalised to the average pixel intensity of the inner circle. All statistical analyses were performed using either Prism 4 or Prism 6 (Graphpad Software, CA USA). Final images and grey scale adjustments were prepared in Adobe Photoshop.

## Additional Information

**How to cite this article**: Grundy, T. J. *et al*. Differential response of patient-derived primary glioblastoma cells to environmental stiffness. *Sci. Rep*. **6**, 23353; doi: 10.1038/srep23353 (2016).

## Supplementary Material

Supplementary Information
