# Supplementary material for: Differential response of patient-derived primary glioblastoma cells to environmental stiffness
Source: Sci Rep. 2016 Mar 21;6:23353. doi: 10.1038/srep23353 (PMC4800394; doi:10.1038/srep23353)
Supplement: Supplementary Information [file srep23353-s1.pdf]

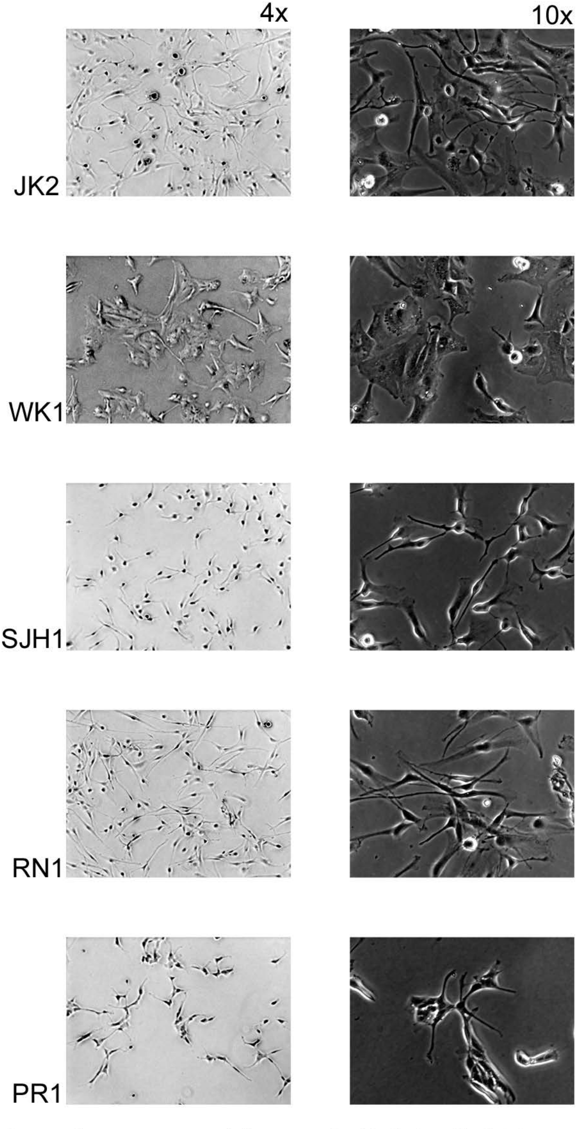

Supplementary Figure 1. Bright field images (4x and 10x magnification) of early passages of the indicated primary GBM cell lines.

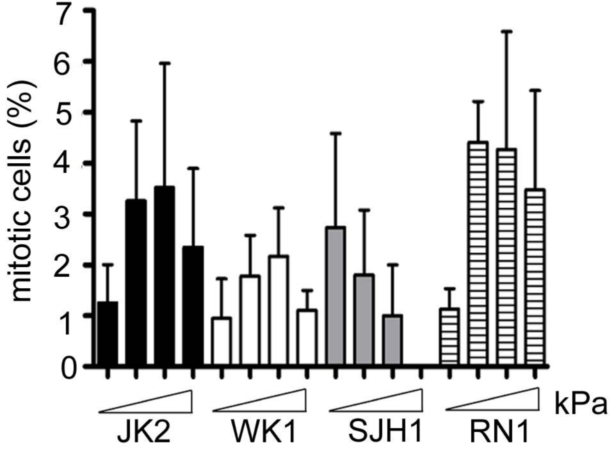

Supplementary Figure 2. The percentage of cells undergoing mitosis throughout the course of the time-lapse videos of the indicated cell lines on the PAM gels of increasing stiffness. There was no significant difference in the percentage of cells in each cell line, one-way ANOVA.

Supplementary Table S1: Characteristics of primary patient-derived glioblastoma (GBM) lines. Molecular subclass was determined by gene expression as previously described[14]. ND = not determined.

Cell line	Sex	Age	Tumour location	Survival (days)	Molecular Subclass
JK2	M	75	right frontal GBM	178	proneural
WK1	M	77	right parieto-occipital GBM	121	mesenchymal
SJH1	M	72	left temporal GBM	45	neural
RN1	M	56	left temporal GBM	243	classical
PR1	M	75	right occipital GBM	523	ND
